# Using restricted factor analysis to select anchor items and detect differential item functioning

**DOI:** 10.3758/s13428-018-1151-3

**Published:** 2018-11-06

**Authors:** Laura Kolbe, Terrence D. Jorgensen

**Affiliations:** Department of Child Development and Education, University of Amsterdam, Amsterdam, The Netherlands

**Keywords:** Factor analysis, Differential item functioning, Latent moderated structural equations, Product indicators, Measurement invariance

## Abstract

Restricted factor analysis (RFA) is a powerful method to test for uniform differential item functioning (DIF), but it may require empirically selecting anchor items to prevent inflated Type I error rates. We conducted a simulation study to compare two empirical anchor-selection strategies: a one-step rank-based strategy and an iterative selection procedure. Unlike the iterative procedure, the rank-based strategy had a low risk and degree of contamination within the empirically selected anchor set, even with small samples. To detect nonuniform DIF, RFA requires an interaction effect with the latent factor. The latent moderated structural equations (LMS) method has been applied to RFA and has revealed inflated Type I error rates. We propose using product indicators (PI) as a more widely available alternative to measure the latent interaction. A simulation study, involving several sample-size conditions and magnitudes of uniform and nonuniform DIF, revealed that PI obtained similar power but lower Type I error rates, as compared to LMS.

In the presence of differential item functioning (DIF), observed differences in composite scores (e.g., scale means) might not represent true differences in the construct that a scale is developed to measure. DIF is formally defined as a violation of measurement invariance (Mellenbergh, 1989):

1 $$ {f}_1\left(X|T=t,V=v\right)={f}_2\left(X|T=t\right) $$

where *X* is a set of observed variables measuring the construct of interest *T*, and *V* is a set of variables other than *T* that potentially violate measurement invariance (e.g., groups defined by sex or ethnicity). Throughout this article, we will use the terms *item* and *indicator* interchangeably to refer to the observed indicators *X* of the construct *T*, and refer to the variable *V* as (potential) violators. Function *f*_1_ is the conditional distribution of *X* given *T* and *V*, and *f*_2_ is the conditional distribution of *X* given *T*. If measurement invariance holds (i.e., *f*_1_ = *f*_2_), the measurement of *T* by *X* is invariant with respect to *V*. If measurement invariance does not hold (i.e., *f*_1_ ≠ *f*_2_), however, the measurement of *T* by *X* functions differently with respect to *V*. A distinction can be made between uniform and nonuniform DIF, where uniform DIF implies that the magnitude of DIF is constant for all levels of the construct *T*, and nonuniform DIF implies that the magnitude of DIF varies with *T*. In different measurement contexts, DIF goes by many other names, such as measurement bias (Oort, 1992), noninvariance (Byrne, Shavelson, & Muthén, 1989), or differential indicator functioning (Kline, 2011, p. 253).

A common method to test for DIF with respect to a grouping variable *V* is *multiple-group confirmatory factor analysis* (MGCFA; Vandenberg & Lance, 2000), in which a measurement model is estimated for each group, and then invariance constraints are imposed on the parameter estimates in order to test whether any items exhibit DIF. Hence, this method requires sufficiently large samples for each group. *Restricted factor analysis* (RFA; Oort, 1992, 1998) is an alternative when sample sizes are small. In RFA models, the potential violator *V* is added to a measurement model as an exogenous variable that is allowed to covary with *T*. *Multiple-indicator multiple-cause* (MIMIC) models (Muthén, 1989) are statistically equivalent to RFA models, but instead of covariance between *V* and *T*, a causal effect of *V* on *T* is modeled. An advantage of RFA over MGCFA is that the division of the sample into subsamples by *V* is not necessary, but RFA also involves an additional assumption—namely, the homogeneity of common and unique factor variances across groups. If this additional assumption holds, RFA has slightly higher power than MGCFA to detect DIF (Barendse, Oort, Werner, Ligtvoet, & Schermelleh-Engel, 2012).

A possible disadvantage of RFA is that it is not readily suited to detect nonuniform DIF. Because nonuniform DIF implies that the magnitude of DIF varies as a function of the common factor *T*, an interaction effect of *T* with *V* on *X* should be estimated. To this end, RFA has been extended with a distribution-analytic approach to model interactions in factor models called *latent moderated structural equations* (LMS; Barendse, Oort, & Garst, 2010). With LMS, the violator *V* should be modeled as a single-indicator latent variable in the RFA model (or MIMIC model; Woods & Grimm, 2011), in order to enable estimation of a latent interaction of *T* with *V*, thus allowing nonuniform DIF to be estimated as the latent-interaction’s effect(s) on the indicator(s). Barendse et al. (2010; Barendse et al., 2012) showed that RFA with LMS generally has high power (89% to 100%) to detect both uniform and nonuniform DIF, except in conditions with a small sample size and small nonuniform DIF. However, severely inflated Type I error rates have been observed (Barendse et al., 2010; Barendse et al., 2012; Woods & Grimm, 2011). This motivated us to find an alternative method for estimating the interaction effect of *T* with *V* on *X* that would provide better control of Type I error rates.

The first aim of the present study was to compare the performance of LMS with that of product indicators (PI; Kenny & Judd, 1984), which is an alternative method to model interactions between latent variables in structural equation models. We aimed to examine whether this method can minimize the inflated Type I error rates obtained with LMS when testing for DIF using RFA models. The PI method has been studied extensively in the general context of modeling latent interactions in structural equation modeling (SEM; Henseler & Chin, 2010; Lin, Wen, Marsh, & Lin, 2010; Little, Bovaird, & Widaman, 2006; Marsh, Wen, & Hau, 2004), but its performance in RFA models to test for nonuniform DIF has not yet been explored. An advantage of PI over LMS is that it can be implemented in any SEM software package, and several methods for calculating product indicators have been automated in the open-source R package semTools (version 0.5-0; Jorgensen, Pornprasertmanit, Schoemann, & Rosseel, 2018). In contrast, testing for nonuniform DIF with RFA models using LMS can only be applied with the commercial SEM software M*plus* (L. K. Muthén & Muthén, 2012). In addition to its limited availability, this software does not provide most traditional SEM fit indices to test for model fit when using LMS estimation. A preliminary study on the use of PI in RFA models suggested that PI and LMS obtain comparable conclusions about whether an item exhibits (non)uniform DIF (Kolbe & Jorgensen, 2018). However, a more extensive simulation study was necessary in order to (dis)confirm the promising performance of the PI method in RFA models for DIF detection.

Methods for testing DIF generally require the selection of anchor items. These items are indicators used to link the scales of the latent construct of interest across groups, and they are assumed to be DIF-free. A common strategy is to use all items other than the studied item as anchors. This strategy leads to a contaminated subset of anchor items when some items other than the studied item exhibit DIF, which in turn leads to problems such as inaccurate item-parameter estimates and an overestimation of the amount of DIF in the test data (W.-C. Wang, 2004). Hence, Woods (2009) argued that the inflated Type I error rates obtained with LMS might be caused by a contaminated subset of anchor items. A simulation study by Woods and Grimm (2011) showed that LMS still resulted in inflated Type I error rates when using an uncontaminated anchor set, which calls into question whether any alternative method might control Type I errors better, given a valid set of anchor items.

The importance of an uncontaminated anchor set for testing DIF provided a second motivation to our study: to investigate practical methods of empirically identifying anchor items when they are not known a priori. Rather than explicitly selecting anchor items, Barendse et al. (2012) applied RFA with LMS in order to test DIF, iteratively accounting for DIF in one indicator at a time. They showed that this brings Type I error rates closer to the nominal level of significance, although some inflation remains. In the present study, we adapted the iterative procedure suggested by Barendse et al. (2012) as an anchor-selection strategy, to be implemented before testing for DIF—that is, iteratively removing indicators from an anchor set initially consisting of all indicators. The iterative procedure can arguably result in large anchor sets, because it begins by assuming all items as anchors and then selects indicators to remove from this anchor set. The potential danger of a larger anchor set is that it generally displays a higher risk of contamination than a smaller anchor set (Kopf, Zeileis, & Strobl, 2015b). Therefore, we contrasted the iterative procedure with the rank-based strategy proposed by Woods (2009). This is an easily implemented forward-selection strategy, in which a limited proportion of all items—those that show the weakest evidence of DIF—are added to the anchor set. A similar strategy has already been applied in MIMIC models (Chun, Stark, Kim, & Chernyshenko, 2016). Woods (2009) recommended that the number of items in the anchor set should be approximately 10% to 20% of the total number.

We will describe both our adaptation of Barendse et al.’s (2012) iterative procedure and Woods’s (2009) rank-based strategy for empirically selecting anchor items in greater detail in a later section. Because both of these empirical anchor-selection strategies involve preliminary tests of DIF, we begin by describing how to test for DIF using RFA models with both LMS and PI. A description of anchor-selection strategies follows, after which we describe two simulation studies: one to compare anchor-selection strategies, and the other to compare latent-interaction models for detecting DIF.

## Detection of DIF using RFA models

The data-generating model for observed continuous scores *x* with potential uniform and nonuniform DIF can be written as follows

2 $$ {\boldsymbol{x}}_j=\boldsymbol{\tau} +\boldsymbol{\uplambda} {t}_j+\boldsymbol{b}{v}_j+\boldsymbol{c}{t}_j{v}_j+\boldsymbol{\delta} {\boldsymbol{\varepsilon}}_j $$

where ***x***_*j*_ is a vector of observed scores, *t*_*j*_ is the common factor *T* score, *v*_*j*_ is the violator variable *V* score, and ***ε***_*j*_ is a vector of the residual scores of subject *j*. The violator variable *V* can be either observed or latent, continuous or categorical,^1^ and it is allowed to covary with the common factor *T*. The model parameters in Eq. 2 include a vector of intercepts (***τ***), a vector of factor loadings (***λ***) on the common factor *T*, a vector of residual factor loadings (***δ***), and vectors of regression coefficients ***b*** and ***c***. The regression coefficients in ***b*** represent the linear effect of the violator *V* on the observed scores ***x***_*j*_, and a nonzero element in ***b*** indicates uniform DIF (i.e., a violation of scalar, or “strong,” invariance). The regression coefficients in ***c*** represent the nonlinear interaction effects of *V* with *T* on ***x***_*j*_, and a nonzero element in ***c*** indicates nonuniform DIF (i.e., a violation of metric, or “weak,” invariance).

When a MGCFA model is fitted to sample data generated under the population described by Eq. 2, ***b*** and ***c*** are not explicitly estimated, but their effects are implicitly captured by virtue of allowing ***τ*** and ***λ***, respectively, to vary across levels of *V*. In contrast, a single-group RFA model for the common factor *T* with observed indicators *X* can be fitted to the data, where the potential violator *V* is added to the model as an exogenous variable. The analytical RFA model resembles the data-generating Eq. 2, but it fixes ***δ*** = 1 for identification. Furthermore, traditional maximum likelihood estimation of an RFA model is complicated by the inability to calculate the product between an observed violator *V* and the latent *T* in order to estimate the nonlinear interaction effects ***c***. LMS has been proposed as a solution to model these nonlinear interaction effects in RFA models (e.g., Barendse et al., 2010), and we have proposed PI as a more widely available alternative method (Kolbe & Jorgensen, 2018), which we investigated more thoroughly in the present study.

In general, uniform and nonuniform DIF can be detected through RFA by comparing the fit of an unconstrained model with the fit of a constrained model. The unconstrained model freely estimates the *b* and *c* parameters for all items studied (i.e., nonanchor items), fixing the *b* and *c* parameters of the anchor items at zero. In the constrained model, the *b* and *c* parameters of a single studied item are additionally fixed at zero. Any potential DIF in the other to-be-studied items is controlled for, because the *b* and *c* parameters of those items are freely estimated in both models. This minimizes the chance of inflated Type I error rates (Woods & Grimm, 2011).

For each studied item, the constraints on the *b* and *c* parameters can be tested simultaneously via model comparison of that item’s constrained model with the unconstrained model. This comparison produces a likelihood ratio test (LRT) statistic, which is distributed as a *χ*^2^ random variable with *df* = 2. A significant LRT statistic indicates that the studied item functions differently with respect to *V*. To reveal whether this DIF is uniform or nonuniform, follow-up tests of the individual *b* and *c* coefficients can be performed using each parameter’s Wald *z* statistic. We focused our investigation only on the omnibus test with *df* = 2 for each studied item.

### Latent moderated structural equations

RFA has most commonly been extended with LMS in order to test items for nonuniform DIF (Barendse et al., 2010; Barendse et al., 2012; see also Woods & Grimm, 2011, about using LMS to test DIF with MIMIC models). The LMS approach to estimate interaction effects of latent variables is a distributional analytic approach available in M*plus* (L. K. Muthén & Muthén, 2012), which implements a maximum likelihood estimation procedure developed especially for the distributional properties of a model that includes product terms among normally distributed latent factors (Klein & Moosbrugger, 2000). In LMS, the joint distribution of indicators is represented as a finite mixture of normal distributions. The mixture distribution function is used in order to obtain maximum likelihood estimates by means of the expectation maximization algorithm (Dempster, Laird, & Rubin, 1977). The LMS approach assumes multivariate normality for all latent exogenous variables. The most common situation for testing invariance is a comparison of two groups (Putnick & Bornstein, 2016), but when the possible violator *V* is a categorical variable, the normality assumption is violated. This violation can be accounted for by using a robust maximum likelihood estimator (Woods & Grimm, 2011). Additional details on how to apply LMS in M*plus* and an example M*plus* script for fitting the RFA model with LMS are provided by Barendse et al. (2012).

**Fig. 1:**
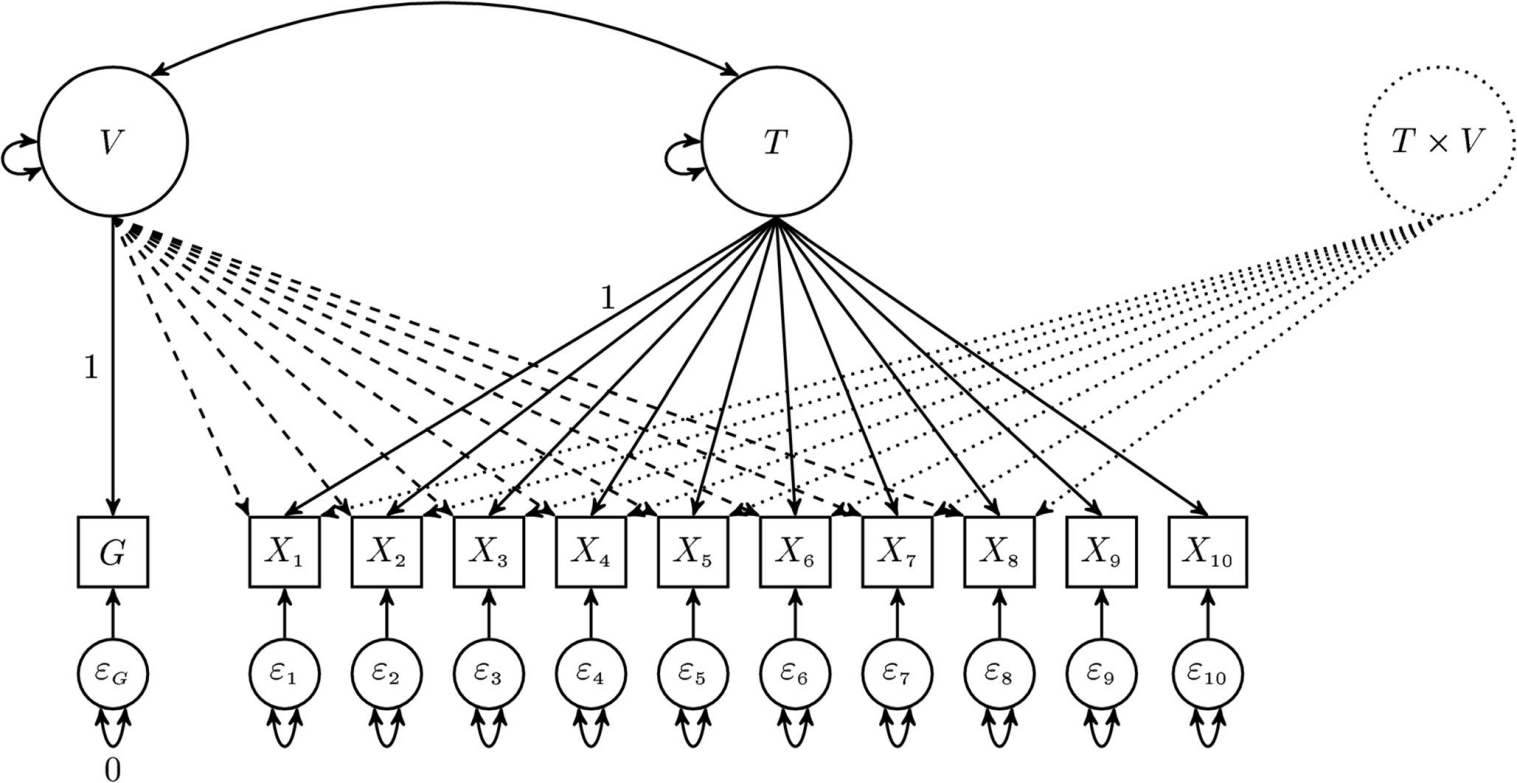
An RFA model with LMS for DIF detection. The items *X*_9_ and *X*_10_ are the anchor items. The dashed and dotted arrows represent effects that may be estimated in order to test for uniform and nonuniform DIF, respectively.

Figure 1 depicts an example RFA model estimable with LMS for DIF detection. The model represents a ten-item case, with the last two items treated as anchors. The LMS approach requires the violator *V* to be modeled as a latent variable. In this example, the violator is measured by a single indicator *G*, representing group membership. As is indicated in Fig. 1, the residual variance of *G* has to be fixed at zero in order for the model to be identified. LMS uses the raw data of all indicators in the model for estimation, but it does not require any indicators of the latent interaction factor *T* × *V*; hence, that factor is represented by a dotted circle. An item can be tested for DIF by comparing the fit of an unconstrained model with the fit of a constrained model. The unconstrained model regresses all studied items on *V* and *T* × *V*, but not the anchor items (in Fig. 1, the items *X*_9_ and *X*_10_ are anchors, not regressed on *V* and *T* × *V*). Put differently, the *b* and *c* parameters only of the anchor items are fixed at zero. In a constrained model, the *b* and *c* parameters of a studied item are additionally set to zero, to test that item for DIF.

### Product indicators

Another possibility for estimating the nonlinear interaction effects in RFA models is the PI method proposed by Kenny and Judd (1984). The PI method involves specifying a measurement model for an additional factor, referred to as the *latent interaction factor*, which represents the interaction between two latent variables. Hence, using the PI method in RFA models requires the violator variable *V* to be modeled as a latent variable, and the measurement model of the latent interaction factor is specified using products between the violator and each of the indicators of *T*. If maximum likelihood is used to estimate the parameters of a model with product indicators, all indicators (including the product indicators) are assumed to be multivariate normally distributed. This assumption is violated, because even the products of normal variables are not normally distributed; the present example, however, involves the product of normal indicators with a binary dummy code, which is itself not normally distributed. A robust maximum likelihood estimator should therefore be used (Marsh et al., 2004).

There are various PI methods that differ in the formation of the product indicators of the latent interaction factor. The most recently proposed PI method is the double-mean-centering strategy (Lin et al., 2010). Using this strategy, indicators for the latent interaction factor are built by mean-centering the product terms produced by multiplying the mean-centered indicators of the associated latent variables. In our ten-item example with a grouping variable as the potential violator, an initial product term between the grouping variable *G* and an indicator (e.g., the first indicator *X*_1_) is first calculated from the mean-centered variables^2^: $\left(G-\overline{G}\right)\left({X}_1-{\overline{X}}_1\right)$. The double mean-centered product indicator is then formed by mean-centering the initial product term: $\left(G-\overline{G}\right)\left({X}_1-{\overline{X}}_1\right)-\overline{\left(G-\overline{G}\right)\left({X}_1-{\overline{X}}_1\right)}$.

Advantages of the double-mean-centering strategy over other strategies are that it does not require that a mean structure be modeled and does not involve a cumbersome multiple-step estimation procedure. An additional advantage is that this strategy outperforms other strategies when the assumption of normality is violated (Lin et al., 2010). See Kolbe and Jorgensen (2018) for an example application of RFA to detect DIF using double-mean-centered product indicators in the R package lavaan (Rosseel, 2012).

**Fig. 2:**
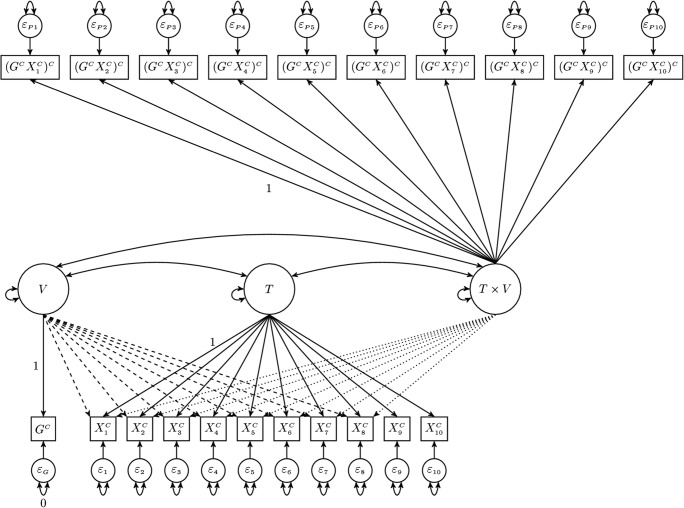
An RFA model with product indicators for DIF detection. The items *X*_9_ and *X*_10_ are the anchor items. The dashed and dotted arrows represent effects that may be estimated in order to test for uniform and nonuniform DIF, respectively.

Figure 2 illustrates the same ten-item example of an RFA model, but the latent interaction factor *T* × *V* is estimated with a measurement model using product indicators calculated via the double-mean-centering strategy. This example includes ten mean-centered indicators, of which the last two are treated as anchors. Each mean-centered indicator of *T* is multiplied by the mean-centered indicator of *V*, and all indicators of *T* × *V* are recentered in order to obtain the double-mean-centered product indicators. The double-mean-centered product indicators are denoted as ${\left({G}^C{X}_k^C\right)}^C$ for the *k* = 1, . . . , 10 items in Fig. 2.

Although the path diagram in Fig. 2 still represents the statistical model fitted to the data, it should not be interpreted as representing an actual data-generating model. The *T* × *V* factor is not an independently identified latent variable, nor are its indicators, so their factor loadings should not be interpreted as the causal effects of *T* × *V* on the product indicators. The product indicators are calculated from other variables in the model, and their loadings merely represent the portion of a product indicator’s variance associated with the product of *T* with *V*, as opposed to the product of *V* with that item’s unique factor. Thus, the “measurement” of a latent *T* × *V* factor is merely an ad hoc technique for extracting the variance that is in common among all of the (double-mean-centered) indicators, so that the effects of the latent *T* × *V* factor on the actual items (i.e., the indicators of *T*) can be estimated in order to detect nonuniform DIF.

As in the LMS method, items can be tested for DIF by comparing the fit of an unconstrained model (i.e., the *b* and *c* parameters of only the anchor items are fixed at zero) with the fit of a constrained model (i.e., the *b* and *c* parameters of the studied item are additionally set to zero). Unlike the ad hoc interpretation of the factor loadings for *T* × *V*, the interpretation of *c*_*k*_ is straightforward: the degree to which the effect of latent factor *T* on observed variable *X*_*k*_ is moderated by *V*.

## Anchor-selection strategies

An anchor-selection strategy guides the decision about which particular items should be used as anchors when testing items for DIF. The anchor items are presumed to be DIF-free and are used to identify the latent construct (i.e., the model would not be identified if all indicators loaded on *T* and were regressed on *V* and *T* × *V*, as well as estimating factor covariances). In RFA models, the anchor items are not regressed on *V* and *T* × *V* when testing studied items for DIF. At least one anchor item is required in order to identify the latent construct in the unconstrained model. Several strategies for selecting or identifying anchor items have been proposed. Some strategies rely on prior knowledge of DIF-free items or on content experts’ advice, whereas empirical strategies are based on preliminary item analysis. This study focused only on empirical anchor-selection strategies. We first describe Woods’s (2009) rank-based strategy, because it involves fewer steps than Barendse et al.’s (2012) iterative procedure.

### Rank-based strategy

The rank-based strategy introduced by Woods (2009) involves a quick and easy procedure to select anchor items. It stems from the idea that the value of each item’s test statistic reflects the magnitude of DIF of that item. The proposed strategy is to test all items for DIF using all other items as anchors. A test statistic with *df =* 2 can be calculated in order to examine DIF for one item at a time. In the context of RFA, the fit of a constrained model can be compared with the fit of several unconstrained models (one per item). In the constrained model, none of the items is regressed on *V* or *T* × *V*, whereas in each unconstrained model, only the studied item is regressed on *V* and *T* × *V*.

After calculating a test statistic for each item’s set of constraints, the items are ranked in an ascending order based on their test statistics. The items with the smallest test statistics (i.e., the weakest evidence of DIF) are selected as anchor items. The actual number of items being selected as anchor items may be determined by factors such as test length and sample size. Woods (2009) suggested that the number of items selected as anchors should be approximately 10%–20% of the total number of items.

### Iterative procedure

The iterative procedure was proposed by Barendse et al. (2012) as a detection method for DIF. However, their procedure can also be applied for the purpose of selecting anchor items (for examples in practice, see Candell & Drasgow, 1988; Hidalgo-Montesinos & Lopez-Pina, 2002; Kopf, Zeileis, & Strobl, 2015a, 2015b). Similar to the rank-based strategy, this procedure involves comparing the fit of a constrained model with the fit of several unconstrained models. In the constrained model, none of the items is regressed on *V* and *T* × *V*, whereas in an unconstrained model, a studied item is regressed on *V* and *T* × *V*. Instead of choosing anchors among the items with the weakest evidence of DIF, all items are initially considered eligible as anchors, and the item with strongest evidence of DIF is removed from consideration.

In the first run of the iterative procedure, the item associated with the largest significant test statistic is considered to function differently. This DIF is taken into account in the second iteration by modifying the constrained and unconstrained models to allow regression of that item on *V* and *T* × *V*. The remaining items are tested for DIF, and again, the item with the strongest evidence of DIF is removed from consideration (assuming the test statistic is significant). The constrained and unconstrained models are again modified by regressing this item on *V* and *T* × *V*, before testing the remaining potential anchor items. This procedure continues until none of the remaining items has a significant test statistic, or until half of the items are considered to function differently. Any remaining items are then considered DIF-free and used as anchor items when testing all other items (again^3^) for DIF.

## Study 1: Selecting anchor items

### Method

In this study, we used simulated data to examine the suitability of the rank-based strategy (Woods, 2009) and the iterative procedure (Barendse et al., 2012) for selecting anchor items. The suitability of these strategies was assessed in the context of extending RFA with both LMS and PI. In addition to the latent-interaction method (LMS vs. PI), we manipulated the anchor-selection strategy (rank-based with 20% or 70% as anchors, or iterative procedure), group sample size (*n* = 50, 100, 150, or 200 per group), and size of DIF (small or large), yielding a 2 × 3 × 4 × 2 factorial design with 1,000 replications in each condition. The relatively small group sample sizes were used because that is the situation when RFA is preferred over MGCFA, which requires larger samples (Oort, 1998). Our outcomes included risk of contamination (i.e., the percentage of replications in which the anchor set contained at least one item exhibiting DIF) and degree of contamination (i.e., the percentage of selected anchor items within each set that exhibited DIF), for which we report the means in each condition.

#### Data generation

Data were generated for two groups under different sample sizes. A scale of *k* = 10 items was considered, of which one item exhibited uniform DIF, one item exhibited nonuniform DIF, and one item exhibited both types of DIF. This allowed us to investigate the performance of the anchor-selection strategies under nonideal conditions because a substantial degree of contamination in the anchor set was possible. The following model was used to generate item scores of subject *j* in group *g*:

3 $$ {\boldsymbol{x}}_j={\boldsymbol{\tau}}_g+{\boldsymbol{\uplambda}}_g{t}_j+{\boldsymbol{\delta}}_g{\boldsymbol{\varepsilon}}_j $$

where ***x***_*j*_ is a vector of ten item scores, *t*_*j*_ is subject *j*’s common factor score, and ***ε***_*j*_ is a vector of residual factor scores of subject *j*. Differences in common factor scores between the groups were simulated by drawing common factor scores from a standard normal distribution for the reference group *t*^*r*^ ∼ *N*(0, 1) and from a normal distribution with a lower mean and variance for the focal group, *t*^*f*^ ∼ *N*(−0.5, 0.7), similar to Barendse et al. (2010). The residual factor scores in both groups were drawn from a standard normal distribution ***ε***_*j*_ ∼ *N*(0, 1).

The group-specific vector ***τ***_*g*_ includes ten intercepts, and **λ**_*g*_ includes ten common factor loadings. We replicated the same magnitude of uniform and nonuniform DIF used by Barendse et al. (2010). Uniform DIF was introduced by imposing across-group differences in intercepts. All intercepts were equal to 0, except for the intercepts for the second and fourth items in the focal group, which were equal to 0.5 in the small-DIF-size conditions and 0.8 in the large-DIF-size conditions. All common factor loadings were equal to 0.8, except for the factor loadings of the third and fourth items in the focal group, which were equal to either 0.55 or 0.3, in the conditions with small and large DIF, respectively. For each group *g*, the vector of residual factor loadings ***δ***_*g*_ was set equal to $\sqrt{1-{\boldsymbol{\lambda}}_g^2}$, so the items had population variances equal to 1.

#### Analytical procedure

Using RFA with both LMS and PI, each item was tested for DIF by comparing the fit of a constrained model with the fit of an unconstrained model. In the constrained model, ***b*** and ***c*** (see Eq. 2) are vectors containing zeros, whereas in the unconstrained model, the elements in ***b*** and ***c*** corresponding to the studied item are freely estimated. The difference in fit between the models was compared using a robust *χ*^2^ statistic with *df* = 2 (Satorra & Bentler, 2010), with *α* = .05 as the criterion for significance. To enable the estimation of the model parameters, group membership was modeled as a latent factor with a single indicator whose factor loading was fixed at unity in each of the models. The residual variance of the group membership indicator was fixed at zero in the RFA models with PI (see Fig. 2), whereas this residual variance was fixed at 0.001 in the RFA models with LMS, to overcome identification problems. For both methods, the common factor *T* was identified by fixing the factor loading of the first item at unity. In RFA models with PI, the factor loading of the first indicator of the interaction factor *T* × *X* was also fixed at unity.

When the iterative procedure was used to select the anchor items, items were iteratively tested for DIF. After each iteration, the item associated with the largest significant *χ*^2^ test statistic was considered to function differently, and this DIF was explicitly modeled in the following iteration. The procedure continued until none of the remaining items was associated with a significant *χ*^2^ statistic or until half (i.e., five) of the items were considered to function differently. Any remaining items, considered DIF-free after the final iteration, were selected for the anchor set. If the *χ*^2^ statistic of one or more of the studied items could not be determined (e.g., because of convergence problems), the procedure was ended and the items considered DIF-free in the previous iteration were selected as the anchor items. With these criteria, the iterative procedure could select 50%–100% of the total number of items as anchors.

With the rank-based strategy, all items were tested for DIF and ranked in ascending order based on their *χ*^2^ statistics. Then, the items with the lowest *χ*^2^ statistics were selected for the anchor set. We examined two versions of the rank-based strategy, one in which 20% of the total number of items were selected as anchor items, as suggested by Woods (2009). To assess the effect of using a larger anchor set, and to compare the results of the rank-based strategy fairly to those of the iterative procedure by having a larger anchor set, we also examined the rank-based strategy when seven items (70% of the total number of items) with the lowest statistics were selected.

In each condition, the risk of contamination was determined, which represented the percentage of replications that yielded an anchor set containing at least one item with DIF. In addition to the risk of contamination, we evaluated the degree of contamination in the anchor set, which was the percentage of items exhibiting DIF in the anchor set. For both risk and degree of contamination, we report the mean across replications in each condition. Because the iterative procedure might result in varying lengths of the anchor sets, the average count (i.e., the average number of items with DIF in the anchor set) was calculated. The RFA models with LMS were fit with M*plus* (version 7; L. K. Muthén & Muthén, 2012) via the R package MplusAutomation (version 0.7; Hallquist & Wiley, 2018). The RFA models with PI were fit with the R package lavaan (version 0.5-23; Rosseel, 2012). The results were analyzed with R (version 3.3.2; R Core Team, 2016).

### Results

After conducting the analysis for each of the conditions, we found that the LMS method did not always produce valid results, due to convergence problems. The percentages of replications with invalid results in each condition with the LMS method are represented in Table 1. Across all conditions, convergence problems occurred in 20.84% of all replications using LMS. The convergence problems did not seem to be associated with either the sample size of the groups or the size of DIF. Among the cases with convergence problems, one or more items could not be tested for DIF because the *χ*^2^ statistic(s) for the corresponding item(s) could not be calculated. When such problems occurred with the LMS method using the rank-based strategy, the results of that replication in that condition were not included in the analysis, because an unambiguous decision about anchor items could not be made in practice. Similarly, when convergence problems occurred with the LMS method in the first run of the iterative procedure, the replication in that condition was not included in the analysis.

**Table 1:** Percentages of replications using latent moderated structural equations (LMS) with invalid results in Study 1

*n*	Percentage of Invalid Results	
	Small DIF	Large DIF
50	23.10	22.50
100	18.00	15.50
150	19.50	17.40
200	24.40	26.30

DIF = differential item functioning. The total number of replications in each condition was 1,000. Only the percentages of invalid results when using LMS are reported in this table, because none of the replications with product indicators obtained invalid results.

In contrast, each of the models converged for every single replication among all conditions using PI. Because the results of the LMS method were based on a smaller number of replications, the validity of comparing results between the two methods could be considered questionable (e.g., if the subsample of replications for which LMS had convergence problems was not a random sample from all 1,000 replications, at least with respect to our outcomes of interest). Therefore, we also calculated results for the PI method using only the replications for which LMS converged. We found the same pattern of results when comparing methods using only the replications that had no convergence problems, so below we present the results using all available converged replications in each condition.

#### Risk of contamination

Table 2 shows the risk and degree of contamination of the selection strategies within each condition. Across all conditions, the rank-based strategy selecting 20% of the total number of items as anchors had the lowest risk of contamination, as compared to the rank-based strategy selecting 70% of the items as anchors and the iterative procedure. The rank-based strategy selecting 20% of the total number of items as anchors had a risk of contamination of 0.00% to 6.11%, whereas the rank-based strategy selecting 70% of the items as anchors had a risk of contamination ranging from 0.40% to 65.70%. The selection strategy with the highest risk of contamination in each of the conditions was the iterative procedure, except in conditions using PI in which the size of DIF was large and the sample size was either 150 or 200. Among all conditions, the iterative procedure had a risk of contamination from 0.20% to 89.50%. The risk of contamination generally decreased with sample size and the size of DIF for each selection strategy. For example, the risk of contamination for the iterative procedure with small DIF and *n* = 200 was less than half of the risk of contamination with small DIF when *n* = 50.

**Table 2:** Results of the anchor-selection strategies for each of the conditions in Study 1

Method	Size of DIF	*n*	Average Risk of Contamination			Average Degree of Contamination (Average Count)		
			RB (20%)	RB (70%)	IP	RB (20%)	RB (70%)	IP
LMS	Small	50	6.11	62.68	88.30	3.06 (0.061)	9.23 (0.646)	14.14 (1.203)
		100	1.46	36.83	73.05	0.73 (0.015)	5.26 (0.368)	11.39 (0.973)
		150	0.62	23.35	55.78	0.31 (0.006)	3.34 (0.234)	9.43 (0.826)
		200	0.13	14.68	43.52	0.07 (0.001)	2.10 (0.147)	8.14 (0.735)
	Large	50	0.52	24.52	52.77	0.26 (0.005)	3.52 (0.247)	8.77 (0.764)
		100	0.12	4.26	26.04	0.06 (0.001)	0.61 (0.043)	5.91 (0.556)
		150	0.00	0.85	17.68	0.00 (0.000)	0.12 (0.008)	4.64 (0.452)
		200	0.95	0.95	20.90	0.47 (0.009)	0.14 (0.009)	5.93 (0.585)
PI	Small	50	5.80	65.70	89.50	2.90 (0.058)	9.66 (0.676)	12.33 (0.995)
		100	2.00	43.40	75.30	1.00 (0.020)	6.20 (0.434)	9.53 (0.753)
		150	0.40	31.80	57.30	0.20 (0.004)	4.54 (0.318)	7.29 (0.573)
		200	0.30	21.30	43.70	0.15 (0.003)	3.04 (0.213)	5.54 (0.437)
	Large	50	2.90	31.80	49.60	1.45 (0.029)	4.63 (0.324)	6.38 (0.506)
		100	0.20	7.30	11.00	0.10 (0.002)	1.04 (0.073)	1.42 (0.111)
		150	0.10	2.20	0.50	0.05 (0.001)	0.31 (0.022)	0.07 (0.005)
		200	0.00	0.40	0.20	0.00 (0.000)	0.06 (0.004)	0.02 (0.002)

The average count (i.e., the average number of DIF items in the anchor set) is reported in parentheses alongside the average degree (as a percentage) of contamination. DIF = differential item functioning; LMS = latent moderated structural equations; PI = product indicators; RB (20%) = rank-based strategy selecting 20% of all items as anchors; RB (70%) = rank-based strategy selecting 70% of all items as anchors; IP = iterative procedure. Risk of contamination = percentage of replications in which the anchor set contained at least one item exhibiting DIF. Degree of contamination = percentage of items exhibiting DIF in the anchor set averaged over all replications

#### Degree of contamination

Similar to the risk of contamination, the degree of contamination typically decreased with sample size and the size of DIF for each selection strategy. The rank-based strategy selecting 20% of the items as anchors had the lowest degree of contamination in the majority of the conditions, with an overall degree of contamination of 0.68%. The only condition in which the rank-based strategy selecting 70% of the items as anchors had a lower degree of contamination was when using LMS with a large size of DIF and a sample size of *n* = 200. In this condition, the rank-based strategy selecting 20% of the items as anchors yielded a degree of contamination of 0.47%, whereas the rank-based strategy selecting 70% as anchor items had a degree of contamination of 0.14%. In all other conditions, the rank-based strategy selecting 20% of the items as anchors performed better than the two other selection strategies with respect to degree of contamination. In addition, the iterative procedure had the highest degree of contamination in the majority of the conditions. To ensure a fair comparison between the anchor-selection strategies on the outcome variables, the average number of anchor items selected by the iterative procedure for each sample size and size of DIF condition is reported in Table 3. The average number of items selected as anchors by the iterative procedure was typically close to seven (ranging from 6.920 to 8.112 across conditions), which was comparable to the number of items selected by the rank-based strategy in the 70% condition.

**Table 3:** Average numbers of items selected as anchors in the iterative procedure

Size of DIF	*n*	Number of Items in the Anchor Set	
		LMS	PI
Small	50	8.112	7.897
	100	7.878	7.671
	150	7.739	7.476
	200	7.631	7.350
Large	50	7.679	7.415
	100	7.460	7.034
	150	7.366	6.929
	200	7.479	6.920

DIF = differential item functioning; LMS = latent moderated structural equations; PI = product indicators. Ideally, only seven items would be included in the anchor set (i.e., the seven items without DIF in the population).

## Study 2: Detecting DIF

### Method

In Study 2, we evaluated the Type I error rates and power of the LMS and PI methods in RFA models to detect uniform and nonuniform DIF. In addition to the latent-interaction method (LMS vs. PI), we again manipulated the reference and focal group sample sizes (*n* = 50, 100, 150, or 200 per group) and the size of DIF (small or large), but not the anchor-selection strategy. Because Study 1 had shown that the rank-based strategy selecting 20% of the total number of items as anchors yielded the lowest risk and degree of contamination in the anchor set, only two out of the ten items were used as anchors in Study 2. However, we manipulated an additional factor (known vs. unknown anchors). The performance of LMS and PI was assessed in the best-case scenario; that is, two known DIF-free items (Items 9 and 10) were used as the anchor items and were not tested for DIF. This best-case scenario always yielded a DIF-free anchor set. By comparison, we also used an empirical-selection scenario, in which the two anchor items selected by the rank-based strategy from Study 1 were used as anchors to test all other items for DIF. This yielded a 2 × 3 × 4 × 2 factorial design, using the same random-number seeds to generate the same 1,000 data sets in each sample-size and DIF-size condition as in Study 1.

#### Analytical procedure

Each item was tested for DIF by comparing the fit of an unconstrained model with the fit of several constrained models (one per studied item) using a robust *χ*^2^ statistic with *df* = 2 (Satorra & Bentler, 2010). In the unconstrained model, all elements in ***b*** and ***c*** were freely estimated, except for the elements corresponding to the anchor items. For all studied items, a constrained model was fitted, in which the corresponding elements in ***b*** and ***c*** for the studied item were fixed at zero. The same identification constraints were used as in Study 1. An item was flagged as an item with DIF when the *χ*^2^ statistic was significant at *α* = .05.

Power and Type I error rates were calculated across all conditions. Power reflects the proportion of replications in which the truly DIF items were correctly flagged as items with DIF. The Type I error rate represents the proportion of replications in which there was at least one Type I error (i.e., one of the DIF-free items was incorrectly flagged as item with DIF). Agresti–Coull confidence intervals^4^ (Agresti & Coull, 1998) around the observed Type I error rates were calculated in order to evaluate the significance of inflation. Power was calculated for each type (uniform, nonuniform, and both) and magnitude (small and large) of DIF separately. The models were fit with M*plus* (version 7; Muthén & Muthén, 2012) via the MplusAutomation package (version 0.7; Hallquist & Wiley, 2018) in the LMS conditions, and with lavaan (version 0.5-23; Rosseel, 2012) in the PI conditions, and the results were analyzed with R (version 3.3.2; R Core Team, 2016).

### Results

After performing the analysis for each of the conditions, we again observed a number of replications with invalid results when using the LMS method. Table 4 shows the percentages of replications with invalid results among the conditions for the best-case and empirical scenarios. On average across all the best-case scenario conditions, invalid results were obtained in 24.39% of all replications using LMS. For each of these replications, the problem involved a nonconverging unconstrained model. Due to this complication, a *χ*^2^ statistic could not be calculated for any of the items. The results of these replications in the best-case scenario were not included in the analysis because, in practice, a researcher would not be able to test for DIF in this situation using RFA. The empirical scenario obtained invalid results in 23.36% of all replications, averaged across the conditions with LMS. These replications were excluded from the analysis for this scenario because, in practice, a decision could not be made regarding the selection of anchor items, or DIF could not be tested for due to a nonconverging unconstrained model.

**Table 4:** Percentages of replications with invalid results in Study 2 for the best-case and empirical scenarios

Method	Size of DIF	*n*	Percentage of Invalid Results	
			Best-Case	Empirical
LMS	Small	50	21.50	24.40
		100	18.10	19.70
		150	26.50	22.50
		200	31.40	26.40
	Large	50	21.50	24.10
		100	18.00	18.30
		150	25.60	22.60
		200	32.50	28.90

DIF = differential item functioning. The total number of replications in each condition was 1,000. Only the percentages of invalid results when using latent moderated structural equations (LMS) are reported in this table, because none of the replications with product indicators obtained invalid results.

The PI method did not produce any convergence problems. All models converged for every replication in each condition. Because the analysis of the LMS method included a smaller number of replications, we again compared results between the two methods using only the replications for which LMS converged. The same pattern of results was found for this smaller set of replications, so we present results using all available converged replications in each condition.

#### Best-case scenario

Table 5 shows the power of LMS and PI across conditions in the best-case scenario (always a DIF-free anchor set). In the majority of the conditions, the PI method obtained a higher power than LMS, although the differences were quite small. Exceptions included the power to detect small nonuniform DIF, which was higher for LMS than for PI. In contrast, large nonuniform DIF was more often detected by PI than by LMS. Power generally increased with sample size for all types and sizes of DIF. Relative to uniform DIF, nonuniform DIF was more difficult to detect, which is consistent with previous research (Barendse et al., 2010). Both LMS and PI especially yielded low power for small nonuniform DIF. With a sample size of *n* = 50, for example, small nonuniform DIF was only detected in 10.80% to 16.20% of all replications. Moreover, the power to detect items exhibiting both uniform and nonuniform DIF was in most conditions comparable to the power to detect uniform DIF.

**Table 5:** Power of the latent moderated structural equations (LMS) and product indicators (PI) methods under each condition of the best-case scenario in Study 2

Type of DIF	*n*	Small DIF		Large DIF	
		LMS	PI	LMS	PI
Uniform	50	.828	.737	.932	.981
	100	.960	.995	.977	1.000
	150	.973	1.000	.991	1.000
	200	.994	1.000	.991	1.000
Nonuniform	50	.162	.108	.535	.464
	100	.341	.218	.834	.839
	150	.544	.358	.882	.973
	200	.672	.493	.825	.996
Combination	50	.660	.710	.925	.977
	100	.947	.994	.966	1.000
	150	.980	1.000	.974	1.000
	200	.993	1.000	.982	1.000

DIF = differential item functioning; small uniform DIF = a difference of 0.5 in intercepts across groups; large uniform DIF = a difference of 0.8 in intercepts across groups; small nonuniform DIF = a difference of 0.25 in factor loadings across groups; large nonuniform DIF = a difference of 0.5 in factor loadings across groups.

Type I error rates for the LMS method in the best-case scenario ranged between .080 and .200 (see Table 6). In each of the conditions, the error rates were significantly larger than the nominal level of significance (5%). The Agresti–Coull confidence intervals for the error rates in each condition with LMS were above the nominal level of significance. The PI method yielded Type I error rates ranging from .047 to .069. When *n* = 100, the error rates were above the nominal level of significance, and the Agresti–Coull lower confidence limits for these error rates were just above the nominal level of significance. In the remaining conditions, the error rates were slightly above or below *α* = .05. However, the error rates for these conditions were not significantly smaller or larger than .05, because the Agresti–Coull confidence intervals for the Type I error rates in the conditions with *n* = 50, *n* = 150, and *n* = 200 included the nominal level of significance. Because there was no reason to expect that only the *n* = 100 condition would yield (barely) inflated error rates, we assumed that this only reflected Monte Carlo sampling error.

**Table 6:** Type I error rates of latent moderated structural equations (LMS) and product indicators (PI) under each condition of the best-case scenario in Study 2

Size of DIF	*n*	Type I Error [95% CI]	
		LMS	PI
Small	50	**.088 [.070, .110]**	.058 [.045, .074]
	100	**.129 [.108, .154]**	**.068 [.054, .085]**
	150	**.151 [.127, .179]**	.051 [.039, .067]
	200	**.197 [.169, .228]**	.047 [.035, .062]
Large	50	**.080 [.063, .101]**	.059 [.046, .075]
	100	**.133 [.111, .158]**	**.069 [.055, .087]**
	150	**.149 [.125, .177]**	.051 [.039, .067]
	200	**.200 [.167, .227]**	.049 [.037, .064]

DIF = differential item functioning. **Bold** font indicates that the lower 95% confidence limit exceeds the nominal 5% alpha level, implying the Type I error rate is statistically significantly inflated. The square brackets contain Agresti–Coull confidence intervals around the error rates.

#### Empirical scenario

Table 7 shows the power of LMS and PI across conditions in the empirical scenario in which two anchor items were selected with the rank-based strategy. The pattern of results found for the empirical scenario was comparable to that in the best-case scenario. For example, similar to the best-case scenario, the PI method had more power to detect DIF than did LMS in the majority of the conditions, but the differences were generally small. Again, a noticeable exception was the power to detect small nonuniform DIF, which was higher for LMS than for PI. With a sample size of *n* = 50, small nonuniform DIF was only detected in 5.70% of all replications using PI.

**Table 7:** Power of the latent moderated structural equations (LMS) and product indicators (PI) methods under each condition of the empirical scenario in Study 2

Type of DIF	*n*	Small DIF		Large DIF	
		LMS	PI	LMS	PI
Uniform	50	.718	.560	.906	.949
	100	.963	.979	.969	.998
	150	.983	1.000	.994	.999
	200	.997	1.000	.992	1.000
Nonuniform	50	.168	.057	.573	.422
	100	.367	.156	.859	.825
	150	.563	.288	.894	.980
	200	.696	.414	.834	.997
Combination	50	.577	.537	.920	.948
	100	.949	.977	.963	.999
	150	.974	.999	.984	1.000
	200	.997	1.000	.997	1.000

DIF = differential item functioning; small uniform DIF = a difference of 0.5 in intercepts across groups; large uniform DIF = a difference of 0.8 in intercepts across groups; small nonuniform DIF = a difference of 0.25 in factor loadings across groups; large nonuniform DIF = a difference of 0.5 in factor loadings across groups.

Type I error rates for the LMS method in the empirical scenario ranged from .077 to .247 (see Table 8). As in the best-case scenario, each of the error rates of LMS was significantly larger than the nominal level of significance. The Agresti–Coull confidence intervals for these error rates were entirely above the nominal level of significance. By comparison, the Type I error rates for the PI method ranged from .022 to .048. In the condition with large DIF and a sample size of *n* = 50 or *n* = 100, the Agresti–Coull confidence interval around the error rate included the nominal level of significance. The confidence intervals of the other conditions were all below *α* = .05.

**Table 8:** Type I error rates of latent moderated structural equations (LMS) and product indicators (PI) under each condition of the empirical scenario in Study 2

Size of DIF	*n*	Type I Error [95% CI]	
		LMS	PI
Small	50	**.077 [.060, .098]**	.022 [.014, .033]
	100	**.105 [.085, .128]**	.026 [.018, .038]
	150	**.129 [.107, .155]**	.023 [.015, .034]
	200	**.247 [.217, .280]**	.035 [.025, .048]
Large	50	**.083 [.065, .105]**	.048 [.036, .063]
	100	**.106 [.087, .130]**	.041 [.030, .055]
	150	**.123 [.101, .148]**	.032 [.023, .045]
	200	**.231 [.201, .263]**	.032 [.023, .045]

DIF = differential item functioning. **Bold** font indicates that the lower 95% confidence limit exceeds the nominal 5% alpha level, implying the Type I error rate is statistically significantly inflated. The square brackets contain Agresti–Coull confidence intervals around the error rates.

## Discussion

The present study concerned testing items for DIF using RFA models. One of the aims of this study was to compare LMS with PI, an alternative method to model latent interactions. We examined whether this method can minimize the inflated Type I error rates obtained with LMS when testing for DIF using RFA models. Woods (2009) argued that the inflated Type I error rates of LMS might be caused by a contaminated set of anchor items. Hence, prior to the comparison between the two methods to model latent interactions, we investigated which anchor-selection strategy is most suitable when testing DIF using RFA models.

The findings of Study 1 indicated that Woods’s (2009) rank-based strategy selecting a small number of items as anchors is more suitable than an iterative procedure of removing items with DIF from the anchor set (Barendse et al., 2012). The rank-based strategy selecting 20% of the total number of items as anchors consistently yielded lower risk and a lower degree of contamination and performed well across all sample sizes. These results are in line with previous studies (M. Wang & Woods, 2017; Woods, 2009), which showed that the rank-based strategy frequently obtains a DIF-free anchor set. The most striking finding of Study 1 was perhaps the high risk of contamination yielded by the rank-based strategy when selecting 70% of the total number of anchor items and by the iterative procedure. These selection strategies allow for larger anchor sets, which generally display a higher risk of contamination than smaller anchor sets (Kopf et al., 2015b). It is also worth noting that other promising empirical anchor-selection strategies have been identified in the item response theory literature that could also generalize well to RFA (or multigroup CFA)—namely, the forward mean test-statistic threshold and forward mean *p*-value threshold methods (Kopf et al., 2015a)—but their implementation is not as straightforward as the rank-based strategy, which yielded excellent results even with small samples. Future research could focus on identifying optimal anchor-selection strategies for factor analysis models in various contexts (e.g., MGCFA).

In Study 2, we compared the LMS and PI methods to model latent interactions in RFA models. The main conclusion was that PI obtained similar power but lower Type I error rates, as compared to LMS. In line with previous studies, severely inflated Type I error rates were observed in conditions with LMS (Barendse et al., 2010; Barendse et al., 2012; Woods & Grimm, 2011). Although it has been argued that the inflated Type I error rates obtained with LMS might be caused by a contaminated set of anchor items (Woods, 2009), our results contradict this possible explanation. The severely inflated error rates were not only observed in the empirical scenario in which contamination of the anchor set was allowed, but also in the best-case scenario with a DIF-free anchor set. This suggests that a contaminated anchor set may not fully account for the frequently observed inflated error rates when using LMS. In response to a reviewer’s suggestion to increase the external validity of our Monte Carlo design, we allowed factor variances to differ across groups. This could explain why our Type I error rates under LMS were larger than those reported by Barendse et al. (2010; Barendse et al., 2012) and Woods and Grimm (2011), given further support by Chun et al.’s (2016) recent demonstration that unequal factor variances yield more inflated Type I error rates than equal factor variances when using LMS.

In contrast, the Type I error rates observed in conditions with PI were all close to the nominal level of significance in the best-case scenario of a DIF-free anchor set, and slightly below the nominal level of significance when using empirically selected anchors. Hence, the results of the present study indicate that the PI method can minimize the inflated Type I error rates obtained with LMS. We suspect a possible explanation for PI’s better control of errors could be the explicitly estimated covariance between the latent factor *T* and the *T* × *V* interaction, which is not a free parameter in LMS estimation algorithms (Klein & Moosbrugger, 2000). This warrants further investigation, but it is beyond the scope of the present investigation.

Corresponding to the findings of previous studies (Barendse et al., 2010; Barendse et al., 2012), we found that nonuniform DIF was more difficult to detect than uniform DIF. Power to detect nonuniform DIF was especially low in conditions with a small sample size. This finding is concerning to some extent, because the present investigation included a best-case scenario of a DIF-free anchor set. As opposed to simulation studies in which the items with true DIF are known, in practice there may seldom be any reliable prior knowledge about DIF in the items of a scale. The results of the empirical scenario, however, showed that empirically selecting anchor items using the rank-based strategy selecting 20% of the total number of items had minor impact on DIF detection. The power to detect DIF using an empirically selected anchor set with this strategy was comparable to the power observed in the best-case scenario. A possible explanation for this minor impact is that the selection strategy used in the empirical scenario had yielded a remarkably low risk and degree of contamination in Study 1. Future research could more extensively investigate the consequences of different anchor-selection strategies on power and Type I error in the context of RFA.

An additional limitation of the LMS method brought to light by the present study is the large proportion of invalid results due to convergence problems. These convergence problems point to an important practical limitation of the LMS method, because in practice they would prevent a researcher from making a decision about anchor items or testing items for DIF. Moreover, this study showed that the PI method to model latent interactions in RFA models generally performs at least as well as the LMS method for the purpose of testing DIF. Because RFA extended with LMS can only be applied with the commercial SEM software M*plus* (L. K. Muthén & Muthén, 2012), knowing that PI is a viable alternative to LMS can provide more researchers with the opportunity to test for nonuniform DIF using RFA with any SEM software package. However, several aspects of the use of PI are yet unclear—for example, which items should serve as product indicators for the interaction factor. There are various possibilities regarding the formation of product indicators; among others are using only the studied item, only the anchor items, the anchor items and the studied item, or all items (the latter strategy was the one employed in the present study). Although this study showed promising results, more research will be necessary in order to determine the optimal use of PI in RFA models to test for DIF.

### Author note

These results were presented as a paper presentation in July 2017, at the 82nd Annual International Meeting of the Psychometric Society (IMPS), in Zürich, Switzerland. We thank Frans Oort for his feedback during the development of the research presented in this article, as well as L. Andries van der Ark, Niels Smits, Dylan Molenaar, Yanyun Yang, and two anonymous reviewers for their feedback on earlier versions of the manuscript. We also thank SURFsara (www.surfsara.nl) for support in using the Lisa Compute Cluster to conduct our Monte Carlo simulations.

## Footnotes

1 Equation 2 could be expanded with additional dummy effects or contrast codes when *V* has > 2 categories. Additional violators could also be added to Eq. 2 to reflect additive or interactive effects on the measurements.

2 The “mean” of a binary dummy code is the proportion of the sample for whom the dummy code equals 1, so the mean-centered dummy code will still have only two levels: the zeros become negative, and the ones become positive. This transformation does not change the interpretation of its effect, because the distance between the negative value and positive value is still one unit. That is, its effect on an indicator is interpreted as the average change in that indicator associated with a one-unit change in the (mean-centered) dummy code, which therefore still represents the difference between two groups’ means (controlling for other predictors, such as *T*). Because the double-mean-centering strategy negates the need for modeling a mean structure, mean-centering a dummy code has no effect on the interpretation of any (covariance structure) model parameters.

3 Recall that DIF is tested for by comparing one constrained model per item to the same unconstrained model (which constrains *b* and *c* only for anchor items). This is distinct from the approach used by the rank-based strategy and iterative procedure, which compare one unconstrained model per item to the same constrained model (which constrains *b* and *c* for all items). The former approach is preferred when testing items for DIF, to prevent inflating Type I error rates due to estimation bias that is introduced by constraining items that exhibit DIF (Woods & Grimm, 2011).

4 Agresti–Coull confidence intervals were obtained by first defining$\overset{\sim }{n}=n+{z}^2$, where *n* is the total number of replications in a single condition, and *z* the 1 − *α*/2 quantile of a standard normal distribution. Then, the midpoint for *X* Type I errors is determined by $\overset{\sim }{p}=\frac{1}{\overset{\sim }{n}}\left(X+\frac{z^2}{2}\right)$. The Agresti–Coull confidence interval around the Type I error rate is given by $\overset{\sim }{p}\pm \sqrt{\frac{\overset{\sim }{p}}{\overset{\sim }{n}}\left(1-\overset{\sim }{p}\right)}$.
